# Sex-based differences in sub-technique selection during an international classical cross-country skiing competition

**DOI:** 10.1371/journal.pone.0239862

**Published:** 2020-09-29

**Authors:** Guro Strøm Solli, Jan Kocbach, Silvana Bucher Sandbakk, Pål Haugnes, Thomas Losnegard, Øyvind Sandbakk

**Affiliations:** 1 Department of Sports Science and Physical Education, Nord University, Bodø, Norway; 2 Centre for Elite Sports Research, Department of Neuromedicine and Movement Science, Faculty of Medicine and Health Sciences, Norwegian University of Science and Technology, Trondheim, Norway; 3 NORCE Norwegian Research Centre AS, Bergen, Norway; 4 Cardiac Exercise Research Group, Department of Circulation and Medical Imaging, Faculty of Medicine and Health Sciences, Norwegian University of Science and Technology, Trondheim, Norway; 5 Department of Physical Performance, Norwegian School of Sports Sciences, Oslo, Norway; Universita degli Studi di Verona, ITALY

## Abstract

The purpose of this study was to compare speed, sub-technique selection and temporal patterns between world-class male and female cross-country (XC) skiers and to examine the combined associations of sex and speed on sub-technique selection. Thirty-three XC skiers performed an international 10-km (women; n = 8) and 15-km (men; n = 25) time-trial competition in the classical style (with the first 10 km of the race being used for analyses). An integrated GNSS/IMU system was used to continuously track position speed and automatically classify sub-techniques and temporal patterns (i.e. cycle length and–rate). When comparing the eight highest ranked men and women, men spent less time than women (29±2 vs. 45±5% of total time) using diagonal stride (DIA), more time (44±4 vs. 31±4%) using double poling (DP) and more time (23±2 vs. 19±3%) using tucking and turning (all *P* < .01). Here, men and women used these sub-techniques at similar temporal patterns within the same speed-intervals; although men employed all sub-techniques at steeper uphill gradients (all *P* < .05). In subsequent analyses including all 33 skiers, adjustment for average racing speed did not fully attenuate the observed sex differences in the proportion of time using DIA (CI_95%_ [-10.7, -1.6]) and DP (CI_95%_ [0.8, 9.3]). Male world-class XC skiers utilized less DIA and more DP compared to women of equal performance levels. Although these differences coincided with men’s higher speed and their ability to use the various sub-techniques at steeper uphill gradients, sexual dimorphism in the proportional use of DIA and DP also occurred independently of these speed-differences.

## Introduction

Cross-country (XC) skiing is performed in two main styles, classical and skating, which are employed while skiing across hilly terrain involving substantial fluctuations in speed and metabolic intensity [1–6]. To manage these varying demands, XC skiers alternate between several sub-techniques specifically adapted to a given terrain and speed as well as the skier’s physical, tactical, and technical abilities [1, 7–10]. Using the classical style, athletes mainly alternate between the diagonal stride (DIA), used at low speeds in uphill terrain, and double poling (DP), which is used at higher speeds on a wide range of inclines [11–14]. In addition, double poling with a kick (DK) is used at moderate speeds when negotiating intermediate inclines; the tuck position is used at the highest speeds in the downhill sections, and various turning techniques are employed in curves [15–17]. Previous analyses have shown that skiers on average alternate between these different sub-techniques 15–25 times per km or 4–6 times per minute, which reflects the sophisticated gearing system used in XC skiing [7, 18, 19].

Although threshold speeds for the usage of the different sub-techniques have been suggested, [7, 18] the constantly changing combinations of speed and inclines during an XC-skiing race complicates the distribution of sub-techniques and related temporal patterns (i.e. cycle length (CL) and–cycle rate (CR)). For example, previous literature has debated the extent to which sub-technique selection is guided by speed [6, 8, 11, 18] or incline [20]. While laboratory experiments provide useful insights into the use of sub-techniques at fixed speed and inclines, a full course analysis is required to obtain deeper insights into the mechanisms underlying individual differences in the choice of sub-technique, the quantity and types of sub-technique transitions, and the temporal patterns within sub-techniques during a real competition.

During an international time-trial competition in the classical style, the observed sex-based difference in average skiing speed was 11% [21]. While this difference is in line with that of many other endurance sports [22], it is likely to have been underestimated in XC skiing due to the longer competition distance for men compared to women (15 km vs. 10 km, respectively). Furthermore, sex differences in XC skiing appear to be dependent on the terrain and sub-technique [7, 23, 24], with an increased sex difference in DP that has increased reliance on power contribution from the upper-body compared to other sub-techniques [25]. This has been explained by the sex-based diversity in upper-body compared to lower-body power [22, 26, 27] and by the fact that men seem to employ more DP and less DIA in the uphill sections than do women during their on-snow training [7]. Video analysis of short sections of intermediate (2–5º incline) terrain further suggests that women are less able to maintain a single technique since they ski closer to their threshold speeds for transitions between sub-techniques during competitions [24]. In these short sections, sex-based differences in DP-cycle characteristics were also present after adjustment for speed [28]. However, sex-based differences in the choice of sub-techniques and temporal patterns have not yet been examined on a full course or among world-class skiers, and it is not known whether differences can be fully explained by the higher speed achieved by men or whether inherent sexual dimorphism is an additional influence.

Wearable sensor technology and the automatic classification of classic sub-techniques using machine-learning algorithms have facilitated field analyses of sub-techniques during XC skiing competitions and training [18, 19, 29–38]. Such information allows more detailed analyses of individual skiers’ choice of sub-technique and thereby provides a deeper understanding of sex differences in the demands of XC skiing. Accordingly, this may help coaches and athletes to more precisely prioritize terrain and choice of sub-techniques in men’s and women’s training. Employing this technology allowed us to compare speed, sub-technique selection, and temporal patterns between male and female world-class XC skiers during the full course of an international time-trial competition utilizing the classical style. We further aimed to investigate the independent and combined associations of sex and speed on sub-technique selection. We hypothesised that men would use the DP technique more frequently than women, particularly in uphill terrain, because of their larger upper-body capacity and ability to obtain higher DP-speeds by longer CL. We expected that these differences would be evident even after adjusting for speed differences between men and women.

## Materials and methods

### Participants

Thirty-three (25 men [age: 24±3 years] and eight women [age: 27±5 years]) world-class XC skiers (29 from Norway and 4 from other countries) competing in an official international FIS-regulated competition volunteered to participate in the study. The analysed competition took place at the start of the competitive season (mid-November) and the skiers were recruited based on their previous performances (i.e. FIS-points) and included in the male and female group based on performance (rank) in this particular competition (see description of matching below). The study was pre-approved by the Norwegian Centre for Research Data, and all participants were fully informed of all test protocols and procedures before they provided their written consent to participate.

### Design

**Fig 1:**
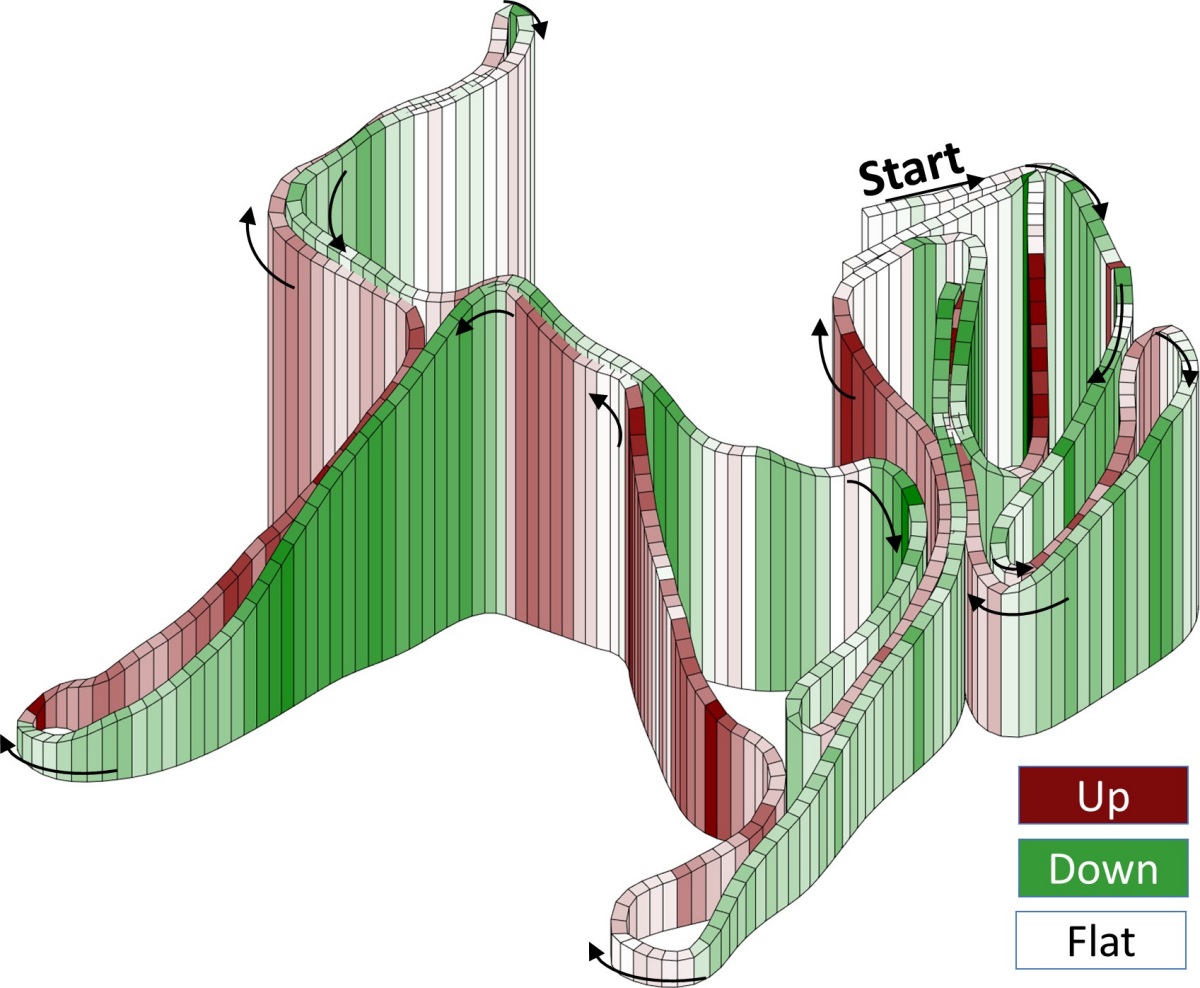
Competition track. 3-dimensional illustration of the 4.6 km competition track examined in the current study.

This study uses an observational, cross-sectional design where performance characteristics during an individual 15-km (men) and 10-km (women) FIS-regulated XC-skiing time-trial competition using the classical technique on snow is examined. Men and women skied three and two laps, respectively, on a 4.7-km track, with a 0.1 km section at the start and finish of each lap being excluded from the analyses (Fig 1). In order to compare men and women over an equal distance, the final lap of the men’s race was excluded from the analyses. For the first analysis, we examined sex-based differences between the eight highest ranked male and female skiers, who were identified based on their FIS points at the time of data collection (13±14 vs. 19±22, *P*>0.5). Their ranking in the analysed competition were 7±4 vs. 8±8 for men and women, respectively (*P*>0.5). All included skiers fulfilled our inclusion criteria of being at a world-class level based on their previous performance (low FIS-points as described above, in combination with regular participation in world-cup competitions over the last two seasons) and having a minimum of ten years of systematic XC ski training. The second part of the analysis examined whether a sex difference in the choice of sub-technique was present also independently of the differences in average race speed between men and women. This was done by including 17 additional male skiers (range of their competition ranking: 15–141) to have overlap of men’s and women’s average race speeds. These were included based on their previous performances (i.e. FIS points ranging from 15 to 125).

All skiers used ski equipment optimised for the specific athlete’s racing preferences, including poles, boots and skis. All ski-base preparations, including grinds, structure, and waxing, were optimised for the snow conditions on the competition day. Men and women participated on the same day with approximately 2 hours in-between. The snow friction and weather conditions in the competitions for men and women were close to identical, with light wind, skies that were partly cloudy, and temperature ranging from -3.3 to -3.6°C. The track was hard-packed artificial snow, which had been machine prepared on the evening before the competition.

### Measurements

Position, speed, and movement data for all athletes were continuously measured using an integrated GNSS and inertial measurement unit (Optimeye S5, Catapult Innovations, Melbourne, Australia), carried in a pocket in the upper part of the race bib. GNSS data is recorded at a 10-Hz sampling frequency and inertial measurement data at a 100-Hz sampling frequency. Velocity is determined based on Doppler shift in the GNSS devices. GNSS lock was ensured by placing the devices in a clear outdoor space for a minimum of 10 minutes prior to data collection to allow for the acquisition of satellite signals. The GNSS devices typically reported 14–17 connected satellites and horizontal dilution of precision (HDOP) of 0.5–0.6 during the data collection. Data was downloaded from the devices on the day of the data collection. The GNSS devices have recently been validated for position, speed and time analysis in XC skiing against a geodetic, multi-frequency receiver by Gløersen and co-workers [39], ensuring that the devices are able to reliably detect differences in performance in line with the research questions in the current study; the horizontal plane position error was 1.04 m (third quartile, Q3) and the precision in horizontal plane speed was 0.072 m/s (IQR), with higher errors at low speeds (i.e. up to 0.36 m/s (Q3) at 2 m/s). The competition course was measured using a high-end differential, multi-frequency and multi-GNSS receiver (Alpha-G3T, Javad, San Jose, CA, United States) to provide a valid course and elevation profile. The skiers’ data were adapted to the standard racecourse for analysis of sub-techniques and temporal patterns in the two first consecutive 4.6-km laps.

### Sub-technique classifications

The course exhibited a varied topography, with a total climb of 154 m. The sub-technique classification was undertaken using a K-Nearest Neighbour algorithm using a sliding window approach with window size of 2 seconds (200 samples) slid by 0.1 seconds. The algorithm was trained on 7 skiers for the racing course used in this competition and 2 skiers for another racing course, and subsequently tested on 3 other skiers with a per-distance classification accuracy of 96% for the complete racing course. The classifications were also examined visually by comparing a graphical representation of filtered accelerometer and gyroscope signals with those typical for the various sub-techniques. Any errors in the automated classification were subsequently corrected based on the visual inspection. In this manner, the sub-techniques were classified as DIA (which included a small amount of the herringbone technique), DK, DP and Other, with the latter including tuck and various turning techniques. The cycles were automatically segmented after classification based on peak detection of Gaussian low pass filtered data from one axis of the gyroscope. Cycle length and time (CT) were determined as the distance or the time, respectively, for each cycle and the cycle speed was calculated by dividing CL by CT. The cycles immediately before and after a sub-technique transition were excluded from the analyses of CL and CR. Cycles involving a transition that lasted <2 seconds were also excluded from the further analyses as they were difficult to definitively categorise. In order to compare world-class male and female skiers over different speed and inclines, speed intervals of ([≤3], (3 to 4], (4 to 5], (5 to 6], (6 to 7], (7 to 8], (8 to 9], (9 to 10], [>10] m/s) and incline intervals of ([≥8], (8 to 6], (6 to 4], (4 to 2], (2 to 0], (0 to-2], (-2 to -4], (-4 to -6], [<-6°) were defined. The speed-incline combination including the highest number of cycles for both men and women for each of the main sub-techniques (DIA [3–4 m/s on 4–6°], DK [4–5 m/s on 2–4°], and DP [5–6 m/s on 0–2°]), were used in the analyses of matched speed and incline-intervals.

### Statistical analysis

Continuous variables are presented as mean±SD and were examined for the assumption of normal distribution prior to analyses using a Shapiro-Wilk test, visual inspection of Q–Q plots and histograms. Categorical variables are presented as absolute numbers and percentages. Significance was set at α = 0.05, and 0.05>α>0.10 was regarded as a trend. Data were processed and analysed using MATLAB 8.1.0. (R2018a; MathWorks Inc., Natick, MA), IBM SPSS Statistics version 24 software for Windows (SPSS Inc., Chicago, IL, USA) and Office Excel 2016 (Microsoft Corporation, Redmond, WA, USA). Pairwise differences in sub-technique selections, transitions and temporal patterns between male and female world-class XC skiers (i.e. the best eight of both sexes) were assessed with independent samples t-tests. Effect size (ES) between groups were calculated according to Cohen’s d, and interpretations of the magnitude were as follows: 0–0.2 = trivial, 0.2–0.6 = small, 0.6–1.2 = moderate, 1.2–2.0 = large, and >2 = very large [40]. Stepwise multiple regression analyses of the full sample (all 33 skiers, i.e. 25 men and eight women) were used to assess independent and combined associations of sex and speed with sub-technique selections. The model fit was examined by normal Q-Q plots of studentized residuals. Residuals were normally distributed as assessed by visual inspection of a normal probability plot.

## Results

### Comparison of world-class male and female XC skiers

**Fig 2:**
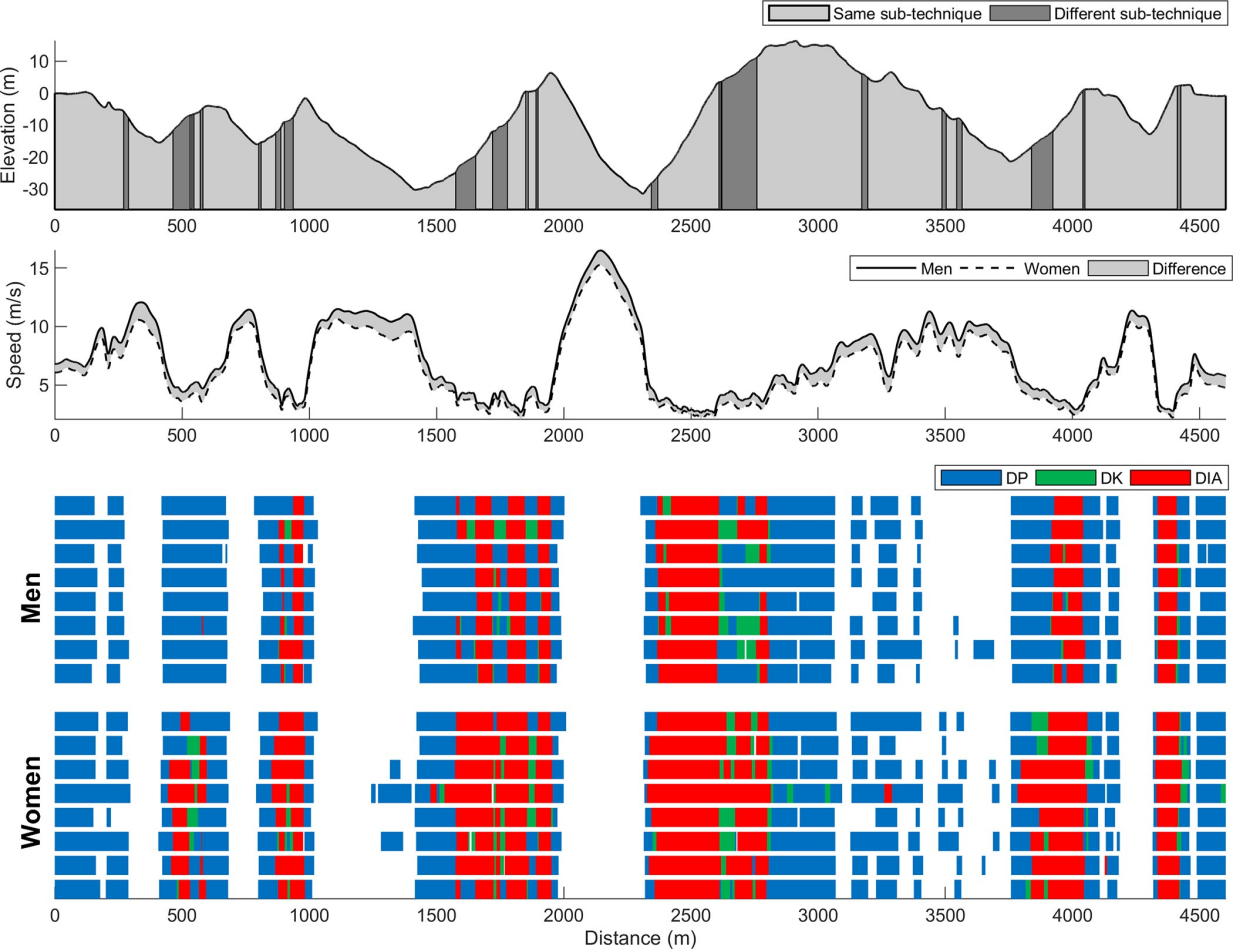
Individual distribution of sub-techniques. Average skiing speed for the eight male and eight female world-class cross-country skiers, and the individual’s use of sub-techniques (diagonal stride [DIA], double poling with a kick [DK], double poling [DP] and other techniques [Other] including tuck and turning techniques) across the racecourse.

The average speeds for men and women, as well as individual use of sub-techniques throughout the course, are presented in Fig 2, whereas the distribution of sub-techniques and temporal patterns are presented in Table 1 and the types of technique transitions in Table 2. The male participants skied, on average 16% faster than females (5.9±0.1 vs. 5.1±0.2 m/s, *P* < .001, ES = 6.41), spent a lower proportion of time using DIA (29±2 vs. 45±5%, *P* < .001, ES = 4.19), but a higher proportion of time using DP (44±4 vs. 31±4%, *P* < .001, ES = 3.45) and Other (23±2 vs. 19±3%, *P* = .006, ES = 1.68). No sex-based difference was found in the proportion of time using DK (4±3 vs. 5±2%, *P* = .432, ES = 0.41). Men performed 13% fewer sub-technique transitions than women (106±12 vs. 122±16, *P* = .049, ES = 1.10), although this difference was not evident when the data was normalised for total race time (Table 2).

**Table 1:** Distribution of sub-techniques and temporal patterns for the eight male and eight female world-class cross-country skiers across the total course (mean±SD).

	DIA				DK				DP				Other			
	Men	Women	*P*	*ES*	Men	Women	*P*	*ES*	Men	Women	*P*	*ES*	Men	Women	*P*	*ES*
Number of cycles	985±86	1728±228	*<* .*001*	*4*.*31*	55±33	82±36	.*142*	*0*.*78*	706±68	616±81	.*030*	*1*.*21*	27±3	30±3	.*077*	*0*.*96*
Time (s)	450±39	810±102	*<* .*001*	*4*.*65*	63±39	91±39	.*175*	*0*.*71*	683±55	569±53	.*002*	*2*.*12*	355±27	345±53	.*635*	*0*.*24*
Distance (m)	1460±160	2437±338	*<* .*001*	*3*.*70*	256±166	351±151	.*253*	*0*.*60*	3839±252	3180±363	.*001*	*2*.*11*	3645±232	3226±425	.*033*	*1*.*22*
Time (%)	29±2	45±5	*<* .*001*	*4*.*19*	4±3	5±2	.*432*	*0*.*41*	44±4	31±4	*<* .*001*	*3*.*45*	23±2	19±3	.*006*	*1*.*68*
Distance (%)	16±2	27±4	*<* .*001*	*3*.*70*	3±2	4±2	.*251*	*0*.*60*	42±3	35±4	.*001*	*2*.*10*	40±3	35±5	.*034*	*1*.*21*
Cycle speed (m/s)	3.2±0.1	3.0±0.1	.*003*	*1*.*96*	4.0±0.2	3.9±0.2	.*120*	*0*.*84*	5.5±0.2	5.5±0.4	.*897*	*0*.*07*	9.3±0.4	8.4±0.4	.*001*	*2*.*24*
Cycle incline (°)	7.4±0.2	6.2±0.3	*<* .*001*	*4*.*12*	3.1±1.1	2.0±0.6	.*028*	*1*.*26*	1.7±0.3	0.4±0.6	*<* .*001*	*2*.*60*	-3.9±0.4	-3.7±0.3	.*401*	*0*.*43*
Cycle length (m)	2.9±0.1	2.8±0.2	.*179*	*0*.*71*	5.1±0.4	4.6±0.3	.*019*	*1*.*34*	5.3±0.2	5.2±0.5	.*105*	*0*.*37*	-	-	*-*	*-*
Cycle rate (Hz)	1.10±0.04	1.07±0.04	.*122*	*0*.*82*	0.80±0.03	0.83±0.04	.*105*	*1*.*03*	1.04±0.06	1.07±0.05	.*346*	*0*.*50*	-	-	*-*	*-*

DIA; diagonal stride, DK; double poling with a kick, DP; double poling, Other; tuck and turning techniques, ES; effect size for the difference between men and women.

**Table 2:** The quantity, speed and incline of the different types of transitions for the eight male and eight female world-class cross-country skiers across the total course (mean±SD).

	#Transitions				Transition speed				Transition incline			
	Men	Women	*P*	*ES*	Men	Women	*P*	*ES*	Men	Women	*P*	*ES*
Total	106.1±11.6	121.5±15.9	.*049*	*1*.*10*	6.0±0.4	5.5±0.3	.*018*	*1*.*34*	0.8±0.5	0.1±0.4	.*015*	*1*.*39*
Total (per minute)	4.1±0.4	4.0±0.4	.*669*	*0*.*22*	-	-	*-*		-	-	*-*	
DP-Other	26.1±2.0	27.6±2.9	.*256*	*0*.*60*	8.2±0.5	7.6±0.5	.*031*	*1*.*20*	-5.3±0.6	-5.7±0.5	.*019*	*0*.*69*
Other-DP	24.5±2.1	27.3±3.5	.*082*	*0*.*95*	7.8±0.4	7.2±0.4	.*007*	*1*.*59*	1.0±0.4	0.6±0.9	.*235*	*0*.*63*
DP-DIA	13.4±3.2	14.0±4.7	.*760*	*0*.*16*	3.7±0.1	3.8±0.4	.*463*	*0*.*38*	6.2±0.5	5.0±0.5	.*001*	*2*.*46*
DIA-DP	12.5±4.0	11.1±5.5	.*578*	*0*.*29*	4.0±0.2	3.7±0.3	.*015*	*1*.*45*	2.5±0.5	1.7±1.0	.*092*	*0*.*94*
DK-DIA	7.9±3.6	10.8±3.9	.*149*	*0*.*76*	3.8±0.2	3.6±0.2	.*068*	*1*.*00*	4.4±0.8	3.1±0.7	.*003*	*1*.*76*
DIA-DK	6.1±3.7	10.1±4.1	.*061*	*1*.*02*	4.0±0.1	3.8±0.2	.*003*	*1*.*76*	2.3±1.3	1.5±0.3	.*112*	*0*.*89*
DK-DP	5.9±3.4	6.6±4.2	.*468*	*0*.*37*	4.5±0.2	4.2±0.3	.*072*	*1*.*13*	1.3±0.5	0.9±1.1	.*189*	*0*.*46*
DP-DK	2.5±2.2	4.5±3.0	.*151*	*0*.*76*	3.9±0.2	3.9±0.4	.*822*	*0*.*12*	4.3±0.8	2.1±0.8	.*001*	*2*.*56*
DIA-Other	-	-	*-*	-	-	-	*-*	-	-	-	*-*	*-*
Other-DIA	-	-	*-*	-	-	-	*-*	-	-	-	*-*	*-*
Other-DK	-	-	*-*	-	-	-	*-*	-	-	-	*-*	*-*
DK-Other	-	-	*-*	-	-	-	*-*	-	-	-	*-*	*-*

DIA; diagonal stride, DK; double poling with a kick, DP; double poling, Other; tuck and turning techniques, ES; effect size for the difference between men and women.

**Fig 3:**
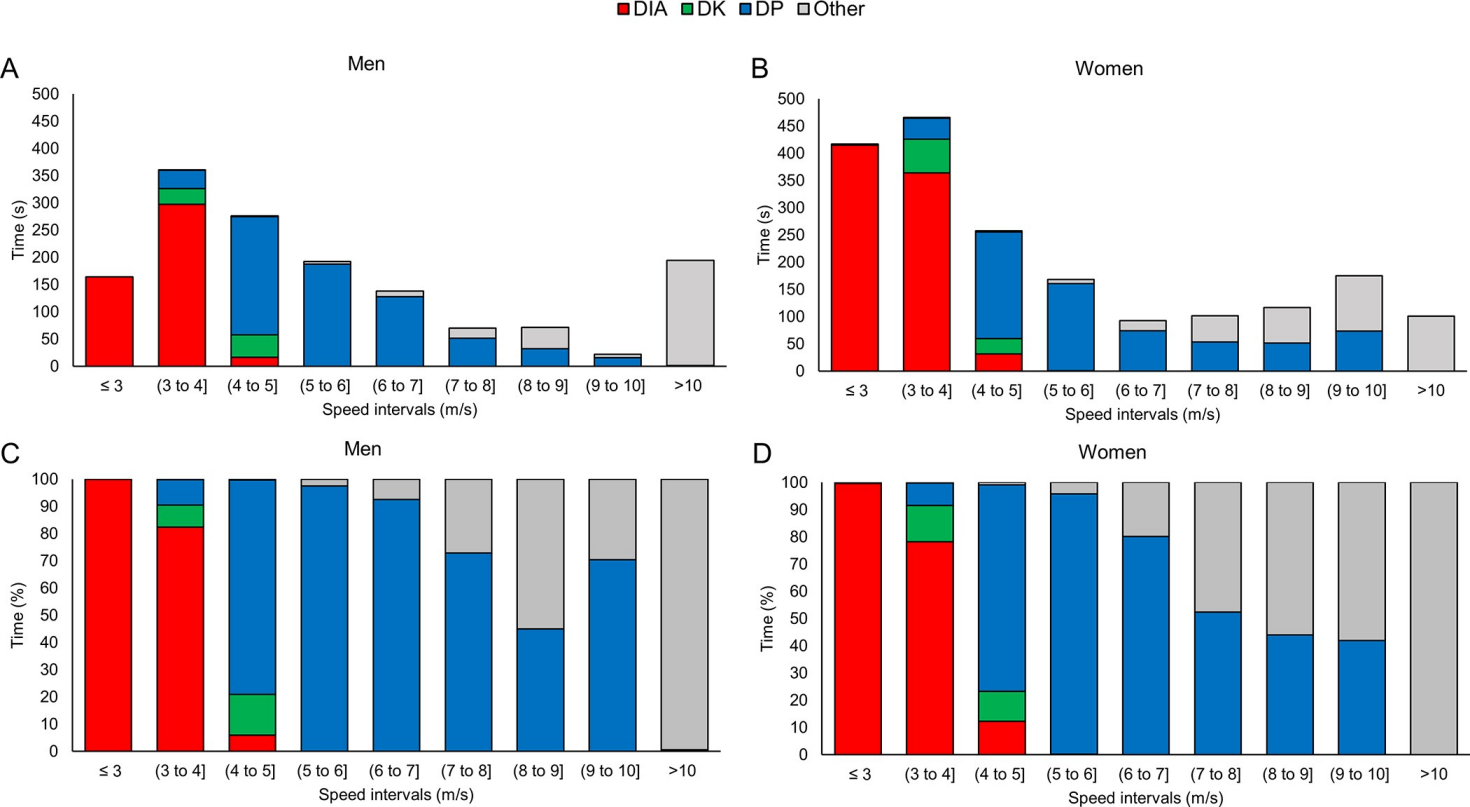
Distribution of sub-techniques over the different speed-intervals. The absolute (A, B) and relative (C, D) distribution of time in each sub-technique (diagonal stride [DIA], double poling with a kick [DK], double poling [DP] and other techniques [Other] including tuck and turning techniques) within different speed-intervals for the eight male and eight female world-class cross-country skiers.

**Fig 4:**
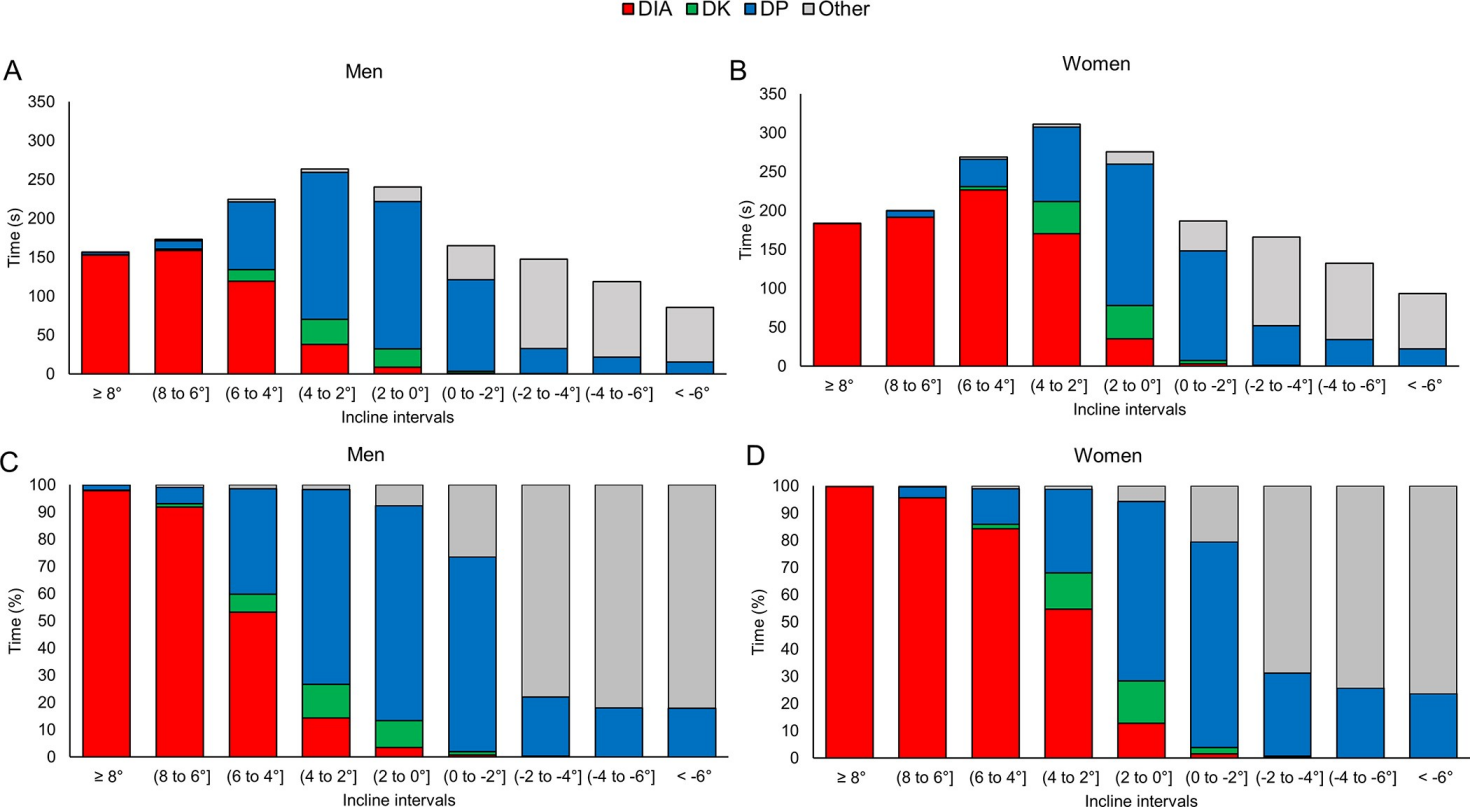
Distribution of sub-techniques over the different incline-intervals. The absolute (A, B) and relative (C, D) distribution of time in each sub-technique (diagonal stride [DIA], double poling with a kick [DK], double poling [DP] and other techniques [Other] including tuck and turning techniques) within different incline-intervals for the eight male and eight female world-class cross-country skiers.

The time spent using each sub-technique within the different speed- and incline-intervals is presented in Figs 3 and 4, respectively (detailed information is available in Supplementary Tables A and B in S1 Data). Both men and women spent the highest proportion of their total race time within speed ranges of 3–4 m/s (23% and 26%, respectively). Men spent less time at speeds <4 and at 7–10 m/s compared to women, but more time at 4–7 and >10 m/s (all *P* < .05, ES>1.3, Fig 3A and 3B). Virtually, there were no differences in the proportion of time of the sub-techniques used within each speed-interval (Fig 3C and 3D).

Both men and women spent the highest proportion of total race time in inclines between 2–4° (both 17%, Fig 4A). Men spent less time and skied faster than women at all incline-intervals (all *P* < .001, ES>3.2). The sex-based differences in speed were 20–21% for intermediate inclines (2–6°), 18–19% for uphill inclines (>6°), 15–17% for flat (2 to -2°) terrain and decreased progressively from 15 to 10% with downhill inclines <0°. Large sex-based differences were observed in the time spent using all sub-techniques within each incline-interval. The most pronounced difference occurred for 2–4°, where men used less DIA (11 vs. 54%) and more DP (77 vs. 31%) compared to women (both *P* < .001, ES>3.6, Fig 4C and 4D).

**Fig 5:**
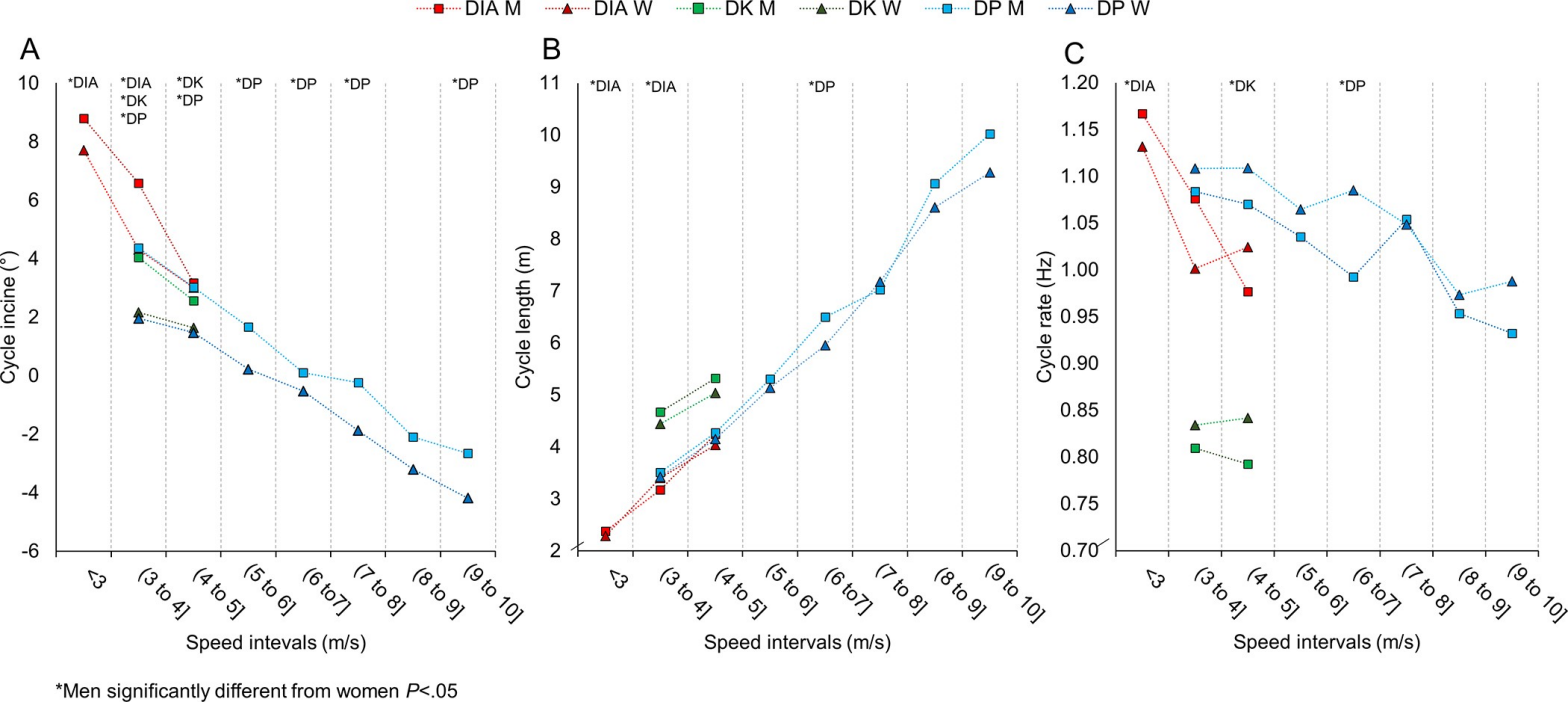
A-C. Kinematic variables over the different speed-intervals. The incline (A), cycle length (B) and cycle rate (C) in each sub-technique (diagonal stride [DIA], double poling with a kick [DK], double poling [DP] and other techniques [Other] including tuck and turning techniques) within different speed-intervals for the eight male and eight female world-class cross-country skiers.

**Fig 6:**
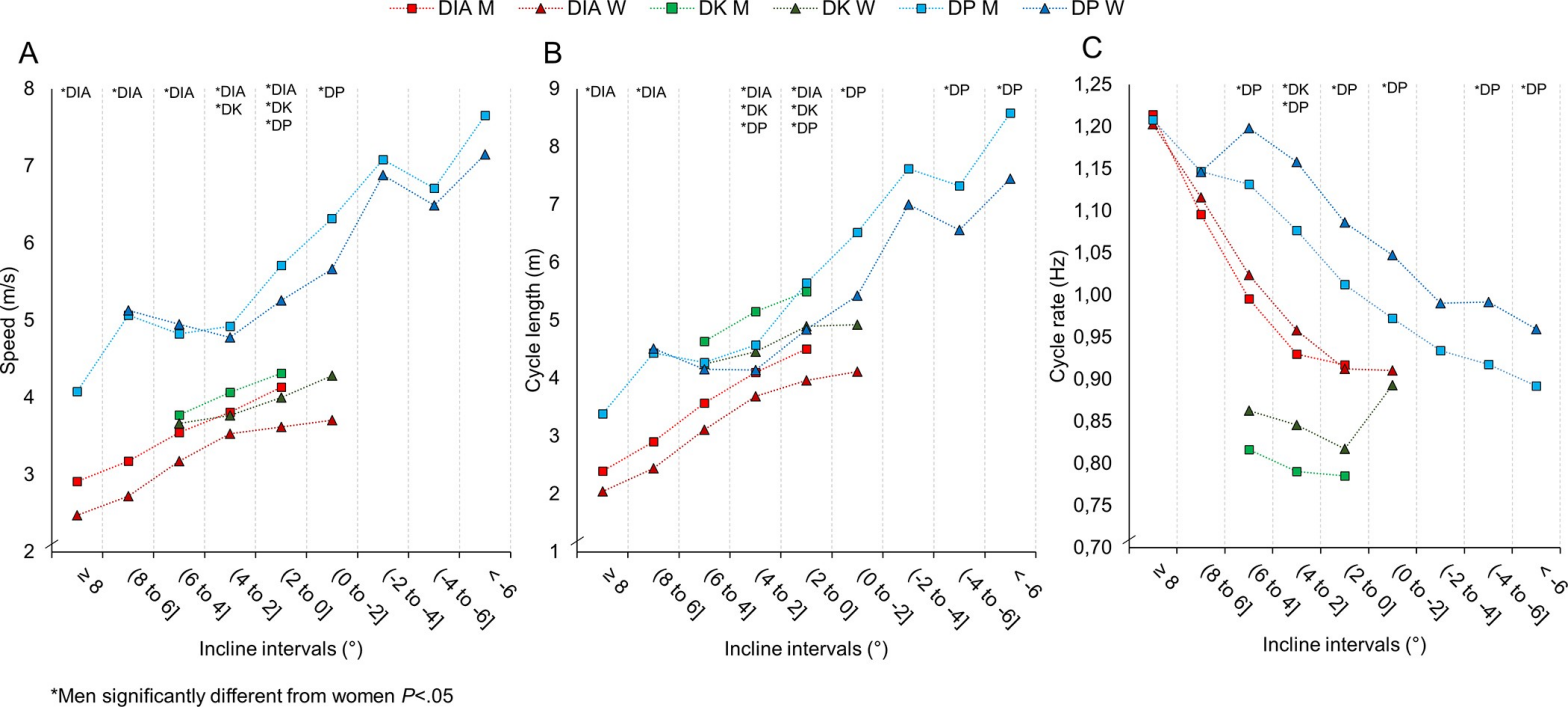
A-C. Kinematic variables over the different incline-intervals. The speed (A), cycle length (B) and cycle rate (C) in each sub-technique (diagonal stride [DIA], double poling with a kick [DK], double poling [DP] and other techniques [Other] including tuck and turning techniques) within different incline-intervals for the eight male and eight female world-class cross-country skiers.

For matched speed-intervals, men generally used DIA, DK, and DP during steeper uphill gradients than women (Fig 5A), whereas the corresponding CL and CR were relatively similar (Fig 5B and 5C). For matched incline-intervals, men generally attained higher cycle speeds for the sub-techniques (Fig 6A), which likely is explained by their longer CL during DIA, and longer CL coupled with slower CR during DK and DP (Fig 6B and 6C). At matched speed-incline combinations, men on average performed 6–9% longer CL (in DIA, DK, and DP) and 6–7% lower CR (in DK and DP) than women (all *P* < .10, ES>0.93).

### Associations between sex, average speed, and the relative use of sub-techniques

**Fig 7:**
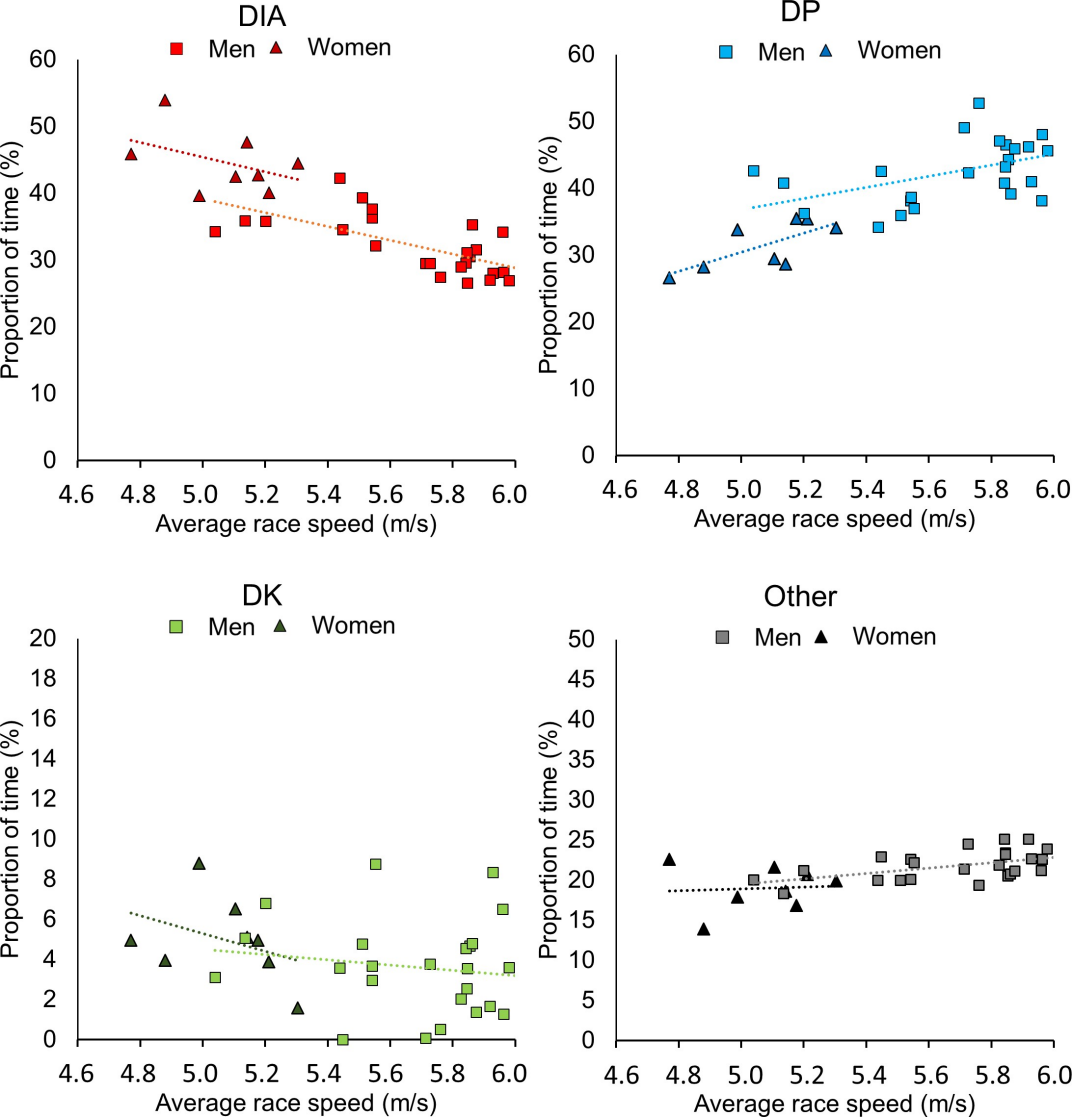
The relationship between the use of sub-techniques and average race speed. The relationship between the proportion of time spent in the different sub-techniques (diagonal stride [DIA], double poling with a kick [DK], double poling [DP] and other techniques [Other] including tuck and turning techniques) and the mean race speed for all 33 (8 women and 25 men) cross-country skiers.

Independent and combined associations between sex, average speed and the proportional use of sub-techniques for all 33 participants are presented in Fig 7. Unadjusted linear regression analyses showed that men spent 12.6%-points less time in DIA (β = -12.6, CI_95%_ [-16.2, -8.9], R^2^ = .62), 11.1%-points more time in DP (β = 11.1, CI_95%_ [7.4, 14.9], R^2^ = .54) and 2.8%-point more time in Other (β = 2.8, CI_95%_ [1.1, 4.6], R^2^ = .27) compared to women over the entire course. No significant sex-based associations were found for DK (β = -1.4, CI_95%_[-3.3, 0.51], R^2^ = .07).

Adjustment for average race speed in these analyses did not fully attenuate the sex difference in the proportional use of sub-techniques, with men spending 6.1%-points less time in DIA (β = -6.1, CI_95%_ [-10.4, -1.8], R^2^ = .76), and 5.6%-points more time in DP (β = 5.6, CI_95%_[0.8, 10.3], R^2^ = .66) indicating that the sex-based difference in sub-technique selection is partly independent of the average race speed.

## Discussion

This study compared speed, sub-technique selection, and temporal patterns between male and female world-class XC-skiers in an international time-trial competition in the classical style, as well as the independent and combined associations of sex and speed on sub-technique selection in a larger sample of elite XC skiers. The major findings related to the comparison of male and female world-class skiers were that: 1) men spent less time using DIA (29% vs. 45%) and more time using DP (44% vs. 31%) and Other (23% vs. 19%, i.e., tuck and turning techniques) than women; and 2) men and women used similar temporal patterns in the various sub-techniques when compared within the same speed intervals, but men employed all sub-techniques at steeper uphill gradients and performed fewer total technique transitions than women. All these results were supported by the strengths of the ES presented. When including 17 more men in the subsequent regression analyses, our final main finding was that part of sexual dimorphism in the proportion of time using DIA and DP was independent of the speed differences between men and women.

This is the first study to investigate the use of sub-techniques and temporal patterns in world-class XC skiers over an entire competition using highly accurate GNSS measurements and automatic technique classification. Comparing men and women over equal distances and terrains (i.e., using the first two 5-km laps of both men’s and women’s races), men spent less time using DIA but correspondingly more time using DP and Other. This is in line with our previous findings in which we investigated sub-technique distribution during low- and high-intensity training in national-level XC skiers [7] and with Stöggl et al. [24], who reported more DP in men than in women during intermediate inclines (2–5°) during the 2016 Norwegian XC skiing championships. The current study expands upon these findings by describing the size of the sex difference in sub-technique selection during a full course among world-class athletes. Here, our analysis showed that women spent approximately 6 minutes more using DIA than men. This has both scientific and practical value, as it provides an enhanced understanding of possible sex differences in the demands of XC skiing and can guide coaches and athletes to select appropriate terrain and sub-techniques in men’s and women’s training.

To provide further insights into sub-technique selection, we analysed how the employment of the different sub-techniques differed across a range of speed and incline intervals. This revealed that the proportion of sub-techniques did not differ between the sexes for given speed intervals (see this illustrated in Fig 3C and 3D). This means that although men skied, on average, 16% faster than women, they used the same proportion of sub-techniques when men and women are compared at the same absolute speed intervals during the racecourse. Accordingly, our data support the existence of speed thresholds for the classical sub-techniques as indicated by previous studies [7, 18, 19] and further suggest that similar threshold speeds apply both to men and women during competitions. This implies that analyses of “speed profiles” (time spent at different speed intervals) of a specific racecourse would provide important information to athletes, coaches and support staff when preparing for a race.

Our analyses of sex-differences across the various incline intervals indicated the largest sex-based difference in skiing speed (20–21%) on intermediate inclines (2–6°), coinciding with a greater sex-based difference in the use of DP vs. DIA on these inclines (Fig 4C and 4D). For example, men spent 77% of their time on intermediate inclines (2–4°) using DP, compared to only 31% for women. In line with this, Stöggl et al. [24] reported the most pronounced sex-based differences in cycle speed (~20%) and a large sex-based diversity in sub-technique selection on intermediate inclines (2–5°). This shows that men’s higher propulsive capacity resulting in higher speed allows for the use of faster sub-techniques on a given incline, which subsequently reinforces the speed differences between men and women on intermediate inclines.

Previous in-competition analyses of CL and CR have typically involved a small window for data collection, using video analysis of sections spanning 12–22 m [10, 24, 28], or reported average values for the entire race [18, 19]. In the current study, we show the development of temporal pattern across the entire competition and have analysed this within different speed and incline intervals. We found that the temporal patterns for men and women were similar across most speed intervals, but that men achieved these speeds at steeper uphill gradients. Accordingly, men and women also used the various sub-techniques at different inclines. For example, men employed DP at steeper gradients than women, with an average cycle incline of 3° for men compared to 1.5° for women at 4–5 m/s, which explains the relatively small sex differences in the temporal patterns for the total course (Table 1). Still, maintaining CL at steeper gradients requires more work per cycle and demonstrates men’s greater potential for generating propulsive force [26, 27]. In our study, this led to particularly pronounced sex-based differences in cycle incline for DP in which the contribution of upper-body power is enlarged [25].

We observed relatively similar CRs within DIA across inclines, which oppose findings by Stöggl et al. [24] who reported 5% lower CR in men than women at uphill inclines. However, men employed lower CR in DP, further substantiating their larger propulsive capacity using this sub-technique. For example, men skied with 17–20% longer CL using DP on flat inclines (–2° to 2°), a finding that corresponds well with the 24% sex-based difference previously observed on flat sections in a competition [28] and the 23% difference observed during maximal roller skiing [25]. In addition, when comparing men and women in cases of matched speed and inclines in the current study, men perform 6–9% longer CL at correspondingly lower CR when using DK and DP. Overall, these findings are in line with previous experiments comparing temporal patterns between men and women in laboratory settings [25, 41].

In this study, we provide novel data on the number of specific types of sub-technique transitions utilized during a XC skiing competition. For both sexes, most of these transitions were between DP and Other (including tuck and turning techniques), which indicates a particular performance-potential to optimize these. In contrast to a previous study [7] in which no sex-based differences in the quantity of sub-technique transitions were observed during training, we observed 13% fewer sub-technique transitions in men than in women when compared over the same distance in an international competition. This is supported by Stöggl et al. [24], who reported fewer transitions in men on intermediate (2–5º) terrain sections and speculated that this was because women spent more time at speeds closer to the speed thresholds for transitions. This could be confirmed by our study, in which women spent more time than men at 3–4 m/s, a speed-interval at which all the main sub-techniques were used, and where the majority of the technique transitions were observed in both sexes. However, we found no sex-based differences when the number of transitions was normalised to the total race time, which indicates that the time spent close to the speed thresholds seems to be the major factor determining the quantity of sub-technique transitions. Therefore, the number of transitions would also be influenced by factors that indirectly influence speed, such as profile of the track, snow conditions, and waxing of skis.

A further aim of this study is to evaluate whether the sex-based differences in sub-technique utilization can be entirely explained by men’s higher skiing speed. Here, we found that some of the sexual dimorphism in the proportional use of DIA and DP was independent of the difference in average race speed. This suggests that men would perform less DIA and more DP than women also when skiing at the same average speed. A likely explanation for this is men’s greater ability to rapidly generate propulsive forces at high speeds during DP (e.g. on the top of hills before entering downhill sections), which results in higher speeds when skiing downhill. Accordingly, the consecutive effect is that men spend more time using DP in cases when uphill sections directly follow downhills.

In general, the abilities to accelerate over hilltops and to maintain speed when entering flat or uphill terrain after downhills are regarded important for winning “easy” seconds during races and should be considered development areas by most skiers. Although previous studies [23, 24] have suggested that women could benefit from “copying men” in such aspects and by increasing their capacity to DP uphill, our findings indicate that the optimal strategy for the use of sub-techniques should differ between men and women. This is likely caused by physiological sex differences [22], which should be taken into consideration by coaches and athletes when developing strategies to improve performance. At least, concurrent development of physical abilities should be considered when striving to improve performance by changing the sub-technique selection during competitions for women.

In contrast to the abovementioned sex-based differences in the employment of DP and DIA, our analysis reveals relatively minor use of DK with no significant sex difference, though there were large individual differences within both sexes. In most cases, DK is used to transition between DP and DIA, and as also observed in previous research, the speed thresholds for DK overlap with those for DIA and DP [7, 18]. Overall, this implies that the use of DK reflects individual preference rather than a general pattern. This is further supported by our finding that there is no association of either sex or speed with the use of DK. Thus, the role and effectiveness of DK are still unclear and are likely multifaceted.

### Methodological considerations

Performing advanced measurements during international competitions are challenging, as the applied sensor technology should not influence athletes’ performance. One of the main strengths of our approach is the application of high-accuracy positioning and IMU measurements, combined with validated algorithms for continuous detection of sub-techniques and temporal patterns during an international on-snow competition. This provides a unique insight into the demands of XC skiing, and specifically on the influence of speed and incline on sub-technique selection and temporal patterns. However, due to the rapid development in sensor technology, more extended analyses of training and competition situations will be possible both in XC skiing and in other outdoor activities in the future. Specifically for XC skiing, the next step in competition analyses would be to develop precise analyses of the fluctuations of external power output, alongside metabolic costs across the different terrain sections. Such analyses would enhance our understanding of optimal pacing strategies, including the impact of friction, air drag and gravity on the ability to generate propulsion across different terrains and sub-techniques. Furthermore, additional analysis of skiers’ physical capabilities could explain to what extent differences between men and women are related to their physical capacities or technical abilities.

Although our analyses in the current study clearly enhance our understanding of the mechanisms underlying sex-differences in XC skiing performance, similar studies should be replicated with other skiers and on different courses and conditions to generalize more conclusively. For example, limitations of the current sex comparison include that men skied 5 km longer than women, that herringbone and DIA could not be distinguished by our algorithm and that a limited of women were included in the regression analysis. In addition, possible sex differences in the length of poles and/or stiffness of skis could have influenced speed and the choice of sub-techniques and temporal patterns across the course. All these aspects might have slightly influenced the size of the sex-differences found here, although it is not likely that these aspects had any impact on our main conclusions.

## Conclusion

In conclusion, male world-class XC skiers utilize less DIA and more DP compared to women at the same performance level. Although these differences coincided with men’s higher speed and ability to use the various sub-techniques at steeper uphill gradients, some of the sexual dimorphism in the proportional use of DIA and DP occurred independently of these speed-differences. A likely explanation for the enlarged use of DP among men is the greater contribution of upper-body power in this sub-technique, in which men is shown particularly superior to women.

## Practical applications

Although the findings in the current study indicate that women could benefit from “copying men” by increasing their capacity to DP on steeper inclines, the optimal strategy for the use of sub-techniques could also differ between men and women. This is an important scientific question to solve in future research, but also vital information for coaches and athletes to understand possible sex differences in the demands of XC skiing and to more goal-oriented prioritize terrain and choice of sub-techniques in men’s and women’s training. Accordingly, future studies should utilize the rapid development in technology and analyses methods to provide further insight into such aspects in XC skiing and other outdoor sports. For example, concurrent analyses of external power and metabolic energy fluctuations of the different competition formats in XC skiing would help our understanding of pacing strategies and enable us to more specifically evaluate how physical, technical and tactical skills influence XC skiing performance.

## Supporting information

S1 Data Overview of the distribution of sub-techniques and kinematic variables over the different speed and incline intervals. (DOCX)

S2 Data Data and analyses conducted in this study. (XLSX)
