# Supplementary material for: Sex-based differences in sub-technique selection during an international classical cross-country skiing competition
Source: PLoS One. 2020 Sep 29;15(9):e0239862. doi: 10.1371/journal.pone.0239862 (PMC7523995; doi:10.1371/journal.pone.0239862)
Supplement: S1 Data — (DOCX) [file pone.0239862.s001.docx]

**Supplementary table A:** Distribution of sub-techniques and kinematic variables (mean±SD^p-value^) for the eight male and eight female world-class cross-country over different speed intervals.

|  | **≤ 3 m/s** | | **(3 to 4] m/s** | | **(4 to 5] m/s** | | **(5 to 6] m/s** | | **(6 to 7] m/s** | | **(7 to 8] m/s** | | **(8 to 9] m/s** | | **(9 to 10] m/s** | | **>10 m/s** | |
| --- | --- | --- | --- | --- | --- | --- | --- | --- | --- | --- | --- | --- | --- | --- | --- | --- | --- | --- |
|  | **M** | **W** | **M** | **W** | **M** | **W** | **M** | **W** | **M** | **W** | **M** | **W** | **M** | **W** | **M** | **W** | **M** | **W** |
| **TOTAL** |  |  |  |  |  |  |  |  |  |  |  |  |  |  |  |  |  |  |
| Time (s) | 146±24 | 417±103^<.001^ | 351±22 | 466±33^<.001^ | 283±13 | 258±14^.002^ | 190±11 | 168±22^.027^ | 143±6 | 93±5^<.001^ | 71±7 | 102±16^.001^ | 82±8 | 105±13^.002^ | 89±11 | 108±12^.005^ | 197±17 | 101±20^<.001^ |
| Time (%) | 9±1 | 23±5^<.001^ | 23±1 | 26±2^.010^ | 18±1 | 14±1^<.001^ | 12±1 | 9±1^<.001^ | 9±0 | 5±0^<.001^ | 5±0 | 6±1^.005^ | 5±1 | 6±1 | 6±1 | 6±1 | 13±1 | 6±1^<.001^ |
| Incline (°) | 8.8±0.1 | 7.6±0.4^<.001^ | 6.0±0.2 | 3.8±0.4^<.001^ | 2.8±0.2 | 1.6±0.2^<.001^ | 1.4±0.1 | -0.1±0.2^<.001^ | -0.3±0.2 | -1.3±0.2^<.001^ | -1.3±0.2 | -2.7±0.2^<.001^ | -2.9±0.2 | -3.4±0.2^<.001^ | -3.3±0.2 | -4.0±0.1^<.001^ | -4.2±0.1 | -4.7±0.2^<.001^ |
| #Transitions | 0±0 | 2±4 | 32±19 | 43±14 | 22±5 | 21±8 | 3±1 | 6±3 | 8±4 | 14±2^.009^ | 13±4 | 17±3^.024^ | 17±4 | 13±5 | 10±4 | 7±4 | 2±2 | 0±1 |
| **DIA** |  |  |  |  |  |  |  |  |  |  |  |  |  |  |  |  |  |  |
| Time (s) | 146±24 | 415±102^<.001^ | 287±36 | 364±67^.016^ | 17±16 | 32±26 |  |  |  |  |  |  |  |  |  |  |  |  |
| Time (%) | 100±0 | 100±1 | 82±7 | 78±10 | 6±6 | 13±11 |  |  |  |  |  |  |  |  |  |  |  |  |
| Cycle incline (°) | 8.8±0.2 | 7.7±0.4^<.001^ | 6.6±0.2 | 4.3±0.4^<.001^ | 3.2±0.9 | 3.0±0.8 |  |  |  |  |  |  |  |  |  |  |  |  |
| Cycle length (m) | 2.4±0.1 | 2.3±0.1^.029^ | 3.2±0.2 | 3.4±0.2^.018^ | 4.2±0.3 | 4.0±0.3 |  |  |  |  |  |  |  |  |  |  |  |  |
| Cycle rate (Hz) | 1.17±0.05 | 1.13±0.04 | 1.08±0.05 | 1.00±0.04^.006^ | 0.98±0.07 | 1.02±0.08 |  |  |  |  |  |  |  |  |  |  |  |  |
| **DK** |  |  |  |  |  |  |  |  |  |  |  |  |  |  |  |  |  |  |
| Time (s) |  |  | 26±14 | 62±31^.013^ | 37±34 | 28±16 |  |  |  |  |  |  |  |  |  |  |  |  |
| Time (%) |  |  | 7±4 | 14±7 | 13±12 | 11±6 |  |  |  |  |  |  |  |  |  |  |  |  |
| Cycle incline (°) |  |  | 4.0±0.5 | 2.2±0.6^.010^ | 2.6±1.1 | 1.6±0.5 |  |  |  |  |  |  |  |  |  |  |  |  |
| Cycle length (m) |  |  | 4.7±0.2 | 4.4±0.2 | 5.3±0.3 | 5.0±0.3 |  |  |  |  |  |  |  |  |  |  |  |  |
| Cycle rate (Hz) |  |  | 0.81±0.04 | 0.83±0.05 | 0.79±0.03 | 0.84±0.04^.018^ |  |  |  |  |  |  |  |  |  |  |  |  |
| **DP** |  |  |  |  |  |  |  |  |  |  |  |  |  |  |  |  |  |  |
| Time (s) |  |  | 38±23 | 38±20 | 227±49 | 195±37 | 186±11 | 161±24^.020^ | 134±12 | 74±9^<.001^ | 53±8 | 53±18 | 31±10 | 39±27 | 13±11 | 7±7 | 1±2 |  |
| Time (%) |  |  | 11±7 | 8±5 | 80±16 | 76±13 | 98±1 | 95±3^.015^ | 93±5 | 80±9^.003^ | 74±8 | 54±18^.016^ | 38±10 | 36±22 | 14±10 | 6±6 | 1±1 |  |
| Cycle incline (°) |  |  | 4.4±0.7 | 2.0±1.2^<.001^ | 3.0±0.3 | 1.5±0.7^<.001^ | 1.7±0.1 | 0.2±0.2^<.001^ | 0.1±0.2 | -0.5±0.5^.004^ | -0.2±0.7 | -1.9±0.6^<.001^ | -2.1±1.4 | -3.2±0.7 | -2.6±0.8 | -4.2±1.0^.010^ |  |  |
| Cycle length (m) |  |  | 3.5±0.2 | 3.4±0.3 | 4.3±0.3 | 4.2±0.3 | 5.3±0.3 | 5.1±0.3 | 6.5±0.5 | 6.0±0.3^.018^ | 7.0±0.4 | 7.2±0.4 | 9.1±0.9 | 8.6±0.6 | 10.0±0.7 | 9.3±0.6 |  |  |
| Cycle rate (Hz) |  |  | 1.08±0.08 | 1.11±0.08 | 1.07±0.08 | 1.11±0.07 | 1.04±0.07 | 1.06±0.06 | 0.99±0.08 | 1.09±0.05^<.012^ | 1.05±0.06 | 1.05±0.05 | 0.95±0.09 | 0.97±0.07 | 0.93±0.05 | 0.99±0.07 |  |  |
| **Other** |  |  |  |  |  |  |  |  |  |  |  |  |  |  |  |  |  |  |
| Time (s) |  |  |  |  |  |  | 4±2 | 7±4 | 9±6 | 19±8^.010^ | 18±6 | 48±26^<.001^ | 51±8 | 65±20 | 76±10 | 102±11^<.001^ | 196±16 | 101±19^<.001^ |
| Time (%) |  |  |  |  |  |  | 2±1 | 4±3^.038^ | 7±5 | 20±9^.003^ | 26±8 | 46±18.^016^ | 62±10 | 64±22 | 86±10 | 94±6 | 100±1 | 100±0^.038^ |
| Cycle incline (°) |  |  |  |  |  |  | -5.3±0.6 | -6.1±0.7 | -6.6±1.4 | -5.6±1.8 | -4.1±1.7 | -3.9±0.9 | -3.1±0.7 | -2.7±0.5 | -3.4±0.6 | -3.3±0.5 | -4.0±0.5 | -5.1±1.3 |
| Cycle length (m) |  |  |  |  |  |  | - | - | - | - | - | - | - | - | - | - | - | - |
| Cycle rate (Hz) |  |  |  |  |  |  | - | - | - | - | - | - | - | - | - | - | - | - |
| DIA; diagonal stride, DK; kick double poling, DP; double pooling, Other; tuck and turning techniques, CL; cycle length, CR; cycle rate. P-vaues P<.05 | | | | | | | | | | | | | | | | | | |

**Supplementary table B:** Distribution of sub-techniques and kinematic variables (mean±SD^p-value^) for the eight male and eight female world-class cross-country over different speed intervals.

|  | **>8°** | | **(8 to 6°]** | | **(6 to 4°]** | | **(4 to 2°]** | | **(2 to 0°]** | | **(0 to -2°]** | | **(-2 to -4°]** | | **(-4 to -6°]** | | **≤ -6°** | |
| --- | --- | --- | --- | --- | --- | --- | --- | --- | --- | --- | --- | --- | --- | --- | --- | --- | --- | --- |
|  | **M** | **W** | **M** | **W** | **M** | **W** | **M** | **W** | **M** | **W** | **M** | **W** | **M** | **W** | **M** | **W** | **M** | **W** |
| Time (s) | 153±3 | 184±13^<.001^ | 170±3 | 200±11^<.001^ | 221±3 | 267±11^<.001^ | 260±3 | 311±12^<.001^ | 235±3 | 276±11^<.001^ | 162±2 | 186±5^<.001^ | 146±2 | 165±4^<.001^ | 118±2 | 132±3^<.001^ | 85±1 | 94±1^<.001^ |
| Time (%) | 10±0 | 10±0 | 11±0 | 11±0 | 14±0 | 15±0^<.001^ | 17±0 | 17±0^.003^ | 15±0 | 15±0 | 10±0 | 10±0^.002^ | 9±0 | 9±0^<.001^ | 8±0 | 7±0^<.001^ | 5±0 | 5±0^<.001^ |
| Speed (m/s) | 2.9±0.1 | 2.5±0.2^<.001^ | 3.4±0.1 | 2.9±0.2^<.001^ | 4.2±0.1 | 3.4±0.1^<.001^ | 4.8±0.1 | 4.0±0.1^<.001^ | 5.9±0.1 | 5.0±0.2^<.001^ | 7.2±0.1 | 6.3±0.2^<.001^ | 9.9±0.1 | 8.7±0.2^<.001^ | 9.8±0.2 | 8.8±0.2^<.001^ | 10.2±0.1 | 9.3±0.1^<.001^ |
| #Transitions | 3±1 | 1±1^<.001^ | 6±3 | 5±1 | 22±4 | 15±8^.028^ | 17±7 | 24±7 | 20±4 | 31±4^<.001^ | 10±2 | 12±4 | 8±3 | 10±3 | 9±2 | 11±3 | 12±3 | 14±2 |
| **DIA** |  |  |  |  |  |  |  |  |  |  |  |  |  |  |  |  |  |  |
| Time (s) | 150±5 | 183±13^<.001^ | 156±4 | 191±11^<.001^ | 110±18 | 227±21^<.001^ | 28±17 | 170±56^<.001^ | 6±5 | 35±20^.004^ | 0±1 | 3±2^.003^ |  |  |  |  |  |  |
| Time (%) | 98±2 | 100±0^<.001^ | 92±2 | 96±1^<.001^ | 50±8 | 85±7^<.001^ | 11±6 | 54±16^<.001^ | 3±2 | 13±7^.004^ | 0±0 | 2±1^.005^ |  |  |  |  |  |  |
| Cycle speed (m/s) | 2.9±0.1 | 2.5±0.2^<.001^ | 3.2±0.0 | 2.7±0.1^<.001^ | 3.5±0.1 | 3.2±0.1^<.001^ | 3.8±0.1 | 3.5±0.1^<.001^ | 4.1±0.1 | 3.6±0.2 | - | 3.7±0.2 |  |  |  |  |  |  |
| Cycle length (m) | 2.4±0.0 | 2.0±0.1^<.001^ | 2.9±0.2 | 2.4±0.2^<.001^ | 3.6±0.3 | 3.1±0.2^.002^ | 4.1±0.2 | 3.7±0.2^.003^ | 4.5±02 | 4.0±0.3 | - | 4.1±0.5 |  |  |  |  |  |  |
| Cycle rate (Hz) | 1.21±0.04 | 1.20±0.04 | 1.10±0.05 | 1.12±0.06 | 1.00±0.06 | 1.02±0.05 | 0.93±0.04 | 0.96±0.04 | 0.92±0.05 | 0.91±0.03 | - | 0.91±0.11 |  |  |  |  |  |  |
| **DK** |  |  |  |  |  |  |  |  |  |  |  |  |  |  |  |  |  |  |
| Time (s) |  |  |  |  | 15±10 | 3±5^.021^ | 29±24 | 41±25 | 17±12 | 43±22^.050^ | 1±1 | 4±3^.038^ |  |  |  |  |  |  |
| Time (%) |  |  |  |  | 7±5 | 1±2^.010^ | 11±9 | 13±8 | 7±5 | 15±8 | 1±1 | 2±2^.048^ |  |  |  |  |  |  |
| Cycle speed (m/s) |  |  |  |  | 3.8±0.1 | - | 4.1±0.2 | 3.8±0.2^.007^ | 4.3±0.2 | 4.0±0.1^.003^ | - | 4.3±0.3 |  |  |  |  |  |  |
| Cycle length (m) |  |  |  |  | 4.6±0.3 | - | 5.2±0.3 | 4.5±0.2^<.001^ | 5.5±0.2 | 4.9±0.2^.001^ | - | 4.9±0.9 |  |  |  |  |  |  |
| Cycle rate (Hz) |  |  |  |  | 0.82±0.04 | - | 0.79±0.04 | 0.85±0.05^.020^ | 0.79±0.03 | 0.82±0.04 | - | 0.89±0.19 |  |  |  |  |  |  |
| **DP** |  |  |  |  |  |  |  |  |  |  |  |  |  |  |  |  |  |  |
| Time (s) | 3±2 | 0±0^<.001^ | 12±2 | 8±1^.005^ | 93±16 | 35±14^<.001^ | 199±32 | 96±30^<.001^ | 192±14 | 182±26 | 117±5 | 141±10^<.001^ | 31±7 | 51±19^.024^ | 21±7 | 34±11^.020^ | 15±7 | 22±10 |
| Time (%) | 2±2 | 0±0^<.001^ | 7±2 | 4±1^.001^ | 42±7 | 13±5^<.001^ | 77±13 | 31±10^<.001^ | 82±6 | 66±11^.005^ | 72±4 | 76±5 | 21±5 | 31±11 | 18±5 | 26±8 | 18±8 | 24±11 |
| Cycle speed (m/s) | 4.1±0.5 | - | 5.1±0.4 | 5.1±0.3 | 4.8±0.1 | 4.9±0.6 | 4.9±0.1 | 4.8±0.3 | 5.7±0.1 | 5.3±0.2^<.001^ | 6.3±0.2 | 5.7±0.2^<.001^ | 7.1±0.3 | 6.9±0.7 | 6.7±0.6 | 6.5±0.5 | 7.7±0.4 | 7.1±0.6 |
| Cycle length (m) | 3.4±0.3 | - | 4.4±0.4 | 4.5±0.5 | 4.3±0.2 | 4.2±0.6 | 4.6±0.2 | 4.1±0.4^.021^ | 5.6±0.3 | 4.8±0.2^<.001^ | 6.5±0.4 | 5.4±0.3^<.001^ | 7.6±0.4 | 7.0±0.7^.050^ | 7.3±0.5 | 6.6±0.6^.012^ | 8.6±0.6 | 7.4±0.5^.001^ |
| Cycle rate (Hz) | 1.21±0.07 | - | 1.15±0.04 | 1.15±0.09 | 1.13±0.06 | 1.20±0.05^.039^ | 1.08±0.07 | 1.16±0.06^.022^ | 1.01±0.07 | 1.09±0.06^.045^ | 0.97±0.08 | 1.05±0.06 | 0.93±0.07 | 0.99±0.05 | 0.92±0.08 | 0.99±0.05 | 0.89±0.05 | 0.96±0.07^.042^ |
| **Other** |  |  |  |  |  |  |  |  |  |  |  |  |  |  |  |  |  |  |
| Time (s) |  |  | 2±1 | 1±0^.007^ | 3±2 | 3±1 | 5±1 | 4±2 | 20±4 | 16±5 | 44±6 | 39±10 | 116±6 | 114±19 | 97±6 | 98±11 | 70±7 | 71±11 |
| Time (%) |  |  | 1±1 | 0±0^.004^ | 2±0 | 1±0 | 2±0 | 1±0 | 8±2 | 6±2^.008^ | 27±4 | 21±5^.019^ | 79±5 | 69±11^.048^ | 82±5 | 74±8 | 82±8 | 76±11 |
| Cycle speed (m/s) |  |  |  |  |  |  |  |  | 7.8±2.1 | 6.5±2.7 | 8.9±0.6 | 8.5±0.7 | 9.6±0.3 | 8.8±0.3^<.001^ | 9.9±0.9 | 8.7±0.6^.010^ | 8.5±0.8 | 6.9±1.1^.004^ |
| Cycle length (m) |  |  |  |  |  |  |  |  | - | - | - | - | - | - | - | - | - | - |
| Cycle rate (Hz) |  |  |  |  |  |  |  |  | - | - | - | - | - | - | - | - | - | - |
| DIA; diagonal stride, DK, kick double poling, DP, double pooling, Other, tuck and turning techniques, CL; cycle length, CR; cycle rate | | | | | | | | | | | | | | | | | | |
